# Apical extrusion of sodium hypochlorite activated with two laser systems and ultrasonics: a spectrophotometric analysis

**DOI:** 10.1186/s12903-015-0056-0

**Published:** 2015-06-26

**Authors:** Bağdagül Helvacıoğlu Kıvanç, Hacer Deniz Arısu, Nazlı Özge Yanar, Hülya Mercan Silah, Recai İnam, Güliz Görgül

**Affiliations:** Gazi University Faculty of Dentistry Department of Endodontics, 8. Cadde 82. Sokak, 06510 Emek-Ankara, Turkey; Gazi University Faculty of Dentistry Department of Restorative Dentistry, Ankara, Turkey; Bilecik Şeyh Edebali University Faculty of Art & Science Department of Chemistry, Bilecik, Turkey; Gazi University Faculty of Art & Science Department of Chemistry, Ankara, Turkey

**Keywords:** Apically extruded irrigating solution, Diode laser, Nd:YAG laser, Passive ultrasonic irrigation, Sodium hypochlorite

## Abstract

**Background:**

The aim of the present study was to compare the effect of ultrasonically or laser (Nd:YAG or diode) activated irrigation on the irrigating solution extrusion compared to non-activated syringe irrigation.

**Methods:**

Extracted mandibular premolar teeth (n = 48) with single canals were instrumented. The teeth were secured through the lid of an Eppendorf tube filled with 1.0 mL distilled water to collect the apically extruded irrigating solution. The teeth were randomly divided into four groups: non-activated syringe irrigation, diode laser, Nd:YAG laser and passive ultrasonic irrigation (PUI) using 2 % NaOCl. The irrigating solution extruded through the apical foramen was collected in the Eppendorf tube and evaluated by a chemical reaction with using a spectrophotometer. The data was analyzed using one-way ANOVA and Tukey’s post hoc test (α = 0.05).

**Results:**

All the groups showed apically extruded irrigating solution. There were significant differences among the groups (p < 0.05). Nd:YAG laser activated irrigation showed greater extrusion (p < 0.05), while the non-activated syringe irrigation showed less extrusion (p < 0.05). Only the difference between diode laser and PUI was not statistically significant (p > 0.05).

**Conclusion:**

Within the limitations of this *in vitro* study, the researchers concluded that non-activated syringe irrigation caused less apically extruded irrigating solution than PUI and LAI using Nd:YAG or diode lasers.

## Background

Root canal irrigation plays an important role in the debridement and disinfection of the root canal system [1]. The goal of irrigation is to remove pulp tissue and/or microorganisms (planktonic or biofilm) from the root canal system [2]. Irrigation is also used to eliminate the smear layer and dentin debris, which occur following instrumentation of the root canal [3]. Sodium hypochlorite at various concentrations is widely used as an endodontic disinfectant thanks to the chemical properties of the pulp dissolution, effective antimicrobial action, dissolution of organic material, transformation of amines into chloramines, and deodorizing effects [4–6].

The mechanical and chemical effectiveness of an irrigation regimen depend on the working mechanisms of the irrigating solution and the ability to bring the irrigating solution in contact with those elements, materials, and structures within the canal system that should be removed [7]. Syringe irrigation is the standard procedure; however, it is not effective in the apical part of the canal [8] and in the isthmus and oval extensions [9]. Moreover, predictable delivery of irrigants to the working length with needle irrigation is not often attained [10]. Nonetheless, some studies suggest that the tip of an injection needle should be placed as close as possible to the apical end of the canal to clean the entire root canal length effectively [10–12]. However, it may increase the risk of extrusion of the solution [13]. As the irrigating solutions are usually cytotoxic, the possibility of an accidental extrusion beyond the apical construction should be considered during root canal irrigation.

Passive ultrasonic irrigation (PUI) was first described by Weller et al. [14]. When a small file or smooth wire placed at the center of the root canal that does not contact the canal wall is activated ultrasonically, ‘acoustic streaming’ occurs. As the root canal is enlarged, the file or wire can vibrate freely in a way to enable acoustic streaming, thereby transferring its energy to the irrigating solution throughout the canal [15]. Various studies have shown that NaOCl used with PUI removes more dentin debris, planktonic bacteria, and pulp tissue from the root canal compared to syringe irrigation [16, 17].

Laser-activated irrigation (LAI) has been introduced as a powerful method for root canal irrigation. Past work has shown that solid-state laser systems with short pulse durations can induce pressure waves in water, including the near-infrared Nd:YAG laser [18] and more recently the middle infrared Er:YAG and Er,Cr:YSGG lasers [19, 20] These laser-generated pressure waves move at high speed, with different characteristics from waves induced by freely vibrating sonic and ultrasonic endodontic instruments [18] and appear to enhance the action of endodontic irrigating solutions in terms of smear layer removal [21]. In the study of Hmud et al. [22] laser-induced cavitations with near-infrared diode lasers was shown in a glass capillary tube model. The laser radiation produces transient cavitation in the liquid through the optical breakdown by absorption of the laser energy. Laser-activated irrigation may result not only in the removal of the smear layer from the root canal wall, but also extrusion of irrigating solution through the apex [20, 21].

The aim of the present study was to compare the effect of ultrasonically or laser (ND:YAG or Diode) activated irrigation on the irrigating solution extrusion compared to non-activated syringe irrigation (control).

The null hypothesis was that the activation methods did not result in more apical extrusion than the control group.

## Methods

This study was approved by Ethics Committee of Ankara University Faculty of Dentistry, Turkey, in accordance with the Declaration of Helsinki (Reference number:36290600/121).

For this study, forty-eight (N = 48) freshly extracted single-rooted human mandibular premolar teeth with single canals were collected from the Clinic of the Department of Oral and Maxillofacial Surgery, Faculty of Dentistry, Gazi University of Ankara, Turkey. The teeth with straight root canals of similar size were included to reduce the effects of canal size and curvature on the extrusion of the irrigant. Radiographic images from the buccal and proximal aspects for each sample were exposed. The teeth with an open apex in the radiographic images were excluded from the study.

Following extraction, the teeth were stored for two days in 3 % NaOCl at room temperature to remove organic debris. They were scaled with ultrasonics, washed with distilled water to remove any calculus or soft tissue debris, and then immersed in 10 % formalin solution until use.

The teeth were decoronated to obtain root segments of 14-mm in length. A 10-K file (Antaeos; Vereinigte Dentalwerke GmbH & Co, Munich, Germany) was placed into the canal until it was visible at the apical foramen. The working length was established as 0.5 mm short of this length.

**Fig. 1:**
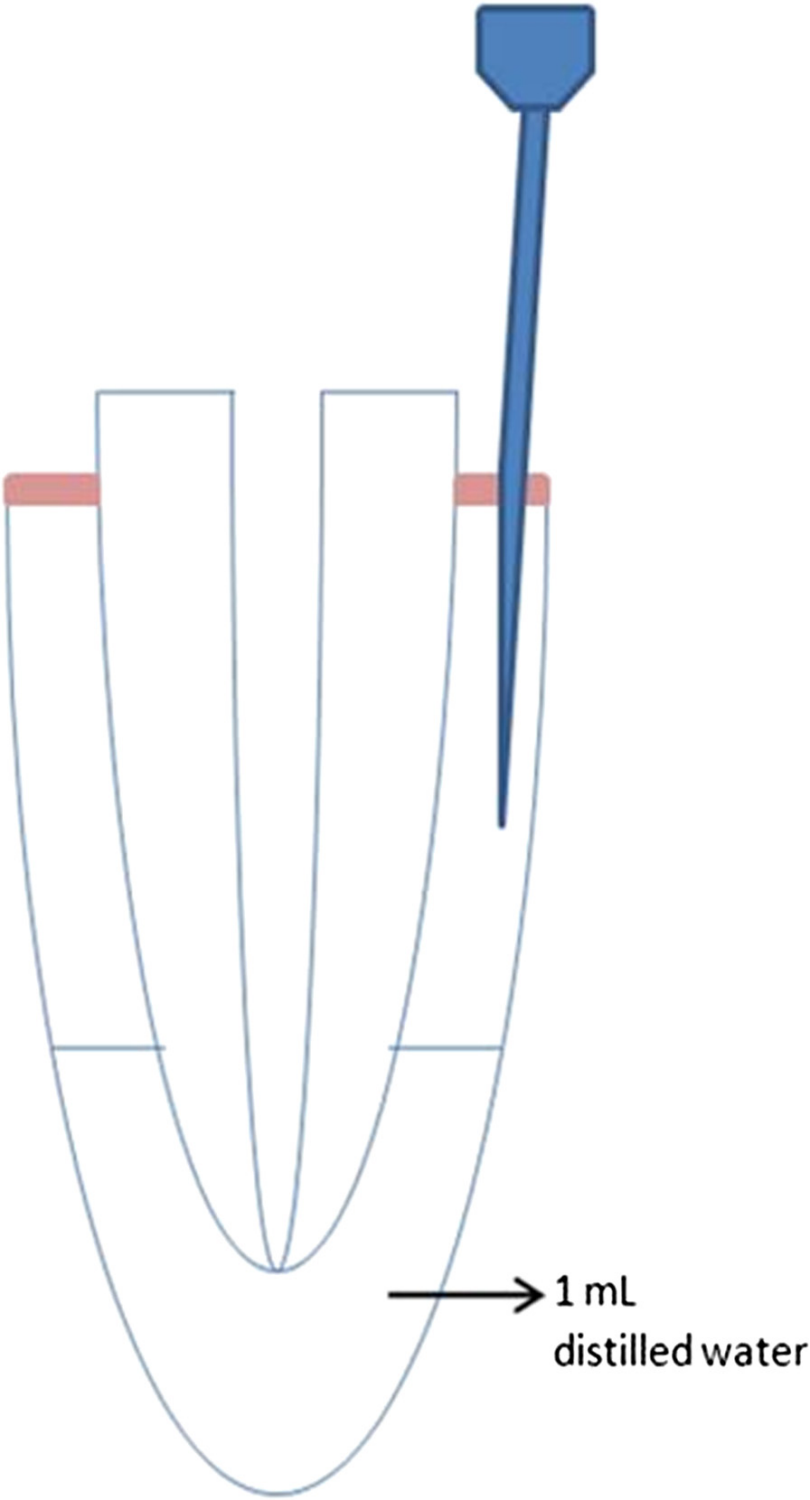
Schematic presentation of the study model

The root canal preparation was performed using rotary instruments (ProTaper, Dentsply, Maillefer, Ballaigues, Switzerland) with a crown down technique. Apical instrumentation was completed with a F3 file (ISO size 30, taper 0.09-0.05). Between the instruments, each canal was irrigated with 2 mL of 2 % NaOCl solution using a syringe and 27-gauge needle. Apical patency was checked with a size 10-K file between each instrument. The teeth were secured with self-curing acrylic resin through the lid of an Eppendorf tube filled with 1.0 mL distilled water to collect the apically extruded irrigating solution. 27-gauge needle was inserted into the Eppendorf tube to equalize the pressure inside and outside the tube (Fig. 1)

The volume of the irrigating solution was kept constant as 4 mL for all control and experimental groups.

Non-activated syringe irrigation (Control group): 4 mL of 2 % NaOCl was injected into the root canals in 60 s with a 27-gauge open-end tip needle, which was placed 2-mm short of the working length without binding (with a constant fluid flow rate of 0.7 mL/sec).

LAI using diode laser (Pocket Diode Laser, Orotig, Verona, Italy): the diode laser (915 nm, 1.2 W, 200 μm fiber optic tip, continuous wave) was activated for a total of 21 s (3 X 7 sec).

LAI using Nd:YAG laser (Pulse Master 600 IQ, Texas, USA): Nd:YAG laser (120 mJ, 20 Hz, 2.4 W, 320 μm fiber optic tip, pulsed mode) was activated for 20 s.

PUI (Satelec, Acteon Group, Merignac, France): PUI was performed with a piezoelectrical ultrasonic unit with power setting 5. A stainless steel size 15-K file (Satalec) was inserted into the root canal and the irrigating solution was ultrasonically activated for 60 s [23].

After LAI using diode laser, LAI using Nd:YAG laser and PUI, the root canals were irrigated with 2 mL of 2 % NaOCl for 30 s with a 27-gauge needle, which was placed 2 mm short of the working length (with a constant fluid flow rate of 0.7 mL/sec).

The quantitiy of hydrochloride ion in an aqueous sample can be determined by finding out how much iodine it can produce by oxidizing an iodide ion [24]. The amount of the extruded NaOCl in the Eppendorf tubes was determined by this chemical reaction of OCl^−^ with I^−^ (iodide) in acidic solution.

$$ \begin{array}{l}\\ {}\begin{array}{ll}\mathbf{2}{\mathbf{e}}^{-} + \mathbf{2}{\mathbf{H}}^{+} + \mathbf{O}\mathbf{C}{\mathbf{l}}^{-}\hfill & \kern0.36em \mathbf{C}{\mathbf{l}}^{-} + {\mathbf{H}}_{\mathbf{2}}\mathbf{O}\hfill \\ {}\mathbf{2}{\mathbf{I}}^{-}\hfill & \kern0.36em {\mathbf{I}}_{\mathbf{2}} + \mathbf{2}{\mathbf{e}}^{-}\hfill \\ {}\mathbf{2}{\mathbf{H}}^{+} + \mathbf{O}\mathbf{C}{\mathbf{l}}^{-} + \mathbf{2}{\mathbf{I}}^{-}\hfill & \kern0.36em {\mathbf{I}}_{\mathbf{2}} + \mathbf{C}{\mathbf{l}}^{-} + {\mathbf{H}}_{\mathbf{2}}\mathbf{O}\hfill \end{array}\end{array} $$

Following the chemical reaction, the amount of NaOCl was determined by spectrophotometric assessment of the change in color due to the formation of iodine (I_2_) in an aqueous solution with a spectrophotometer (Unicam UV2-100 UV/Visible Spectrometer, Aberdeen, UK). The statistical analysis was performed using one-way analysis of variance (ANOVA) and Tukey’s post hoc tests (α = 0.05).

## Results

**Fig. 2:**
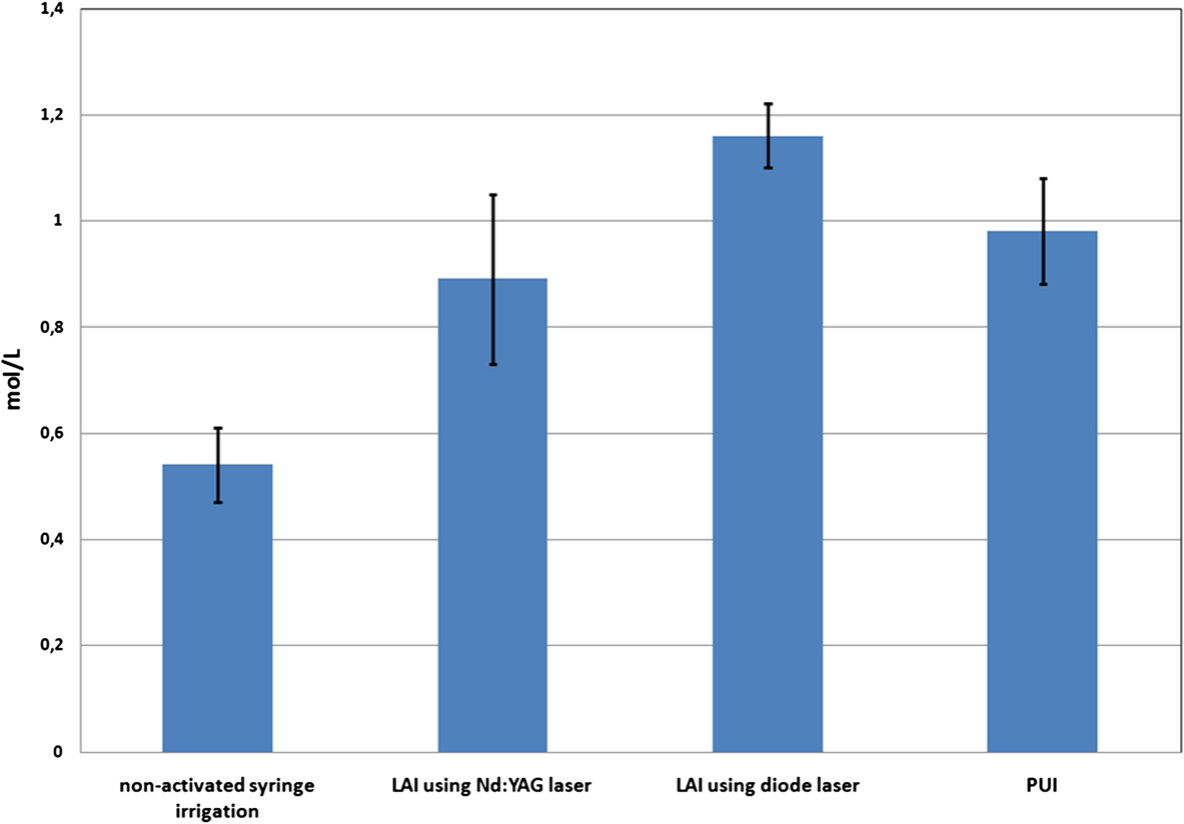
The concentration of apically extruded irrigant (mol/L)

The concentration of the apically extruded irrigating solution is presented in Fig. 2. All the groups showed apical extrusion of irrigating solution. There were significant differences between the groups. (p < 0.05). The non-activated syringe irrigation showed less extrusion, while LAI using Nd:YAG laser showed a greater amount of extrusion. There was only not a significant difference between the LAI using diode laser and PUI (p > 0.05).

## Discussion

With the aim of comparing the effect of the methods used for improving the efficacy of NaOCl irrigation, the researchers assessed more apical extrusion of the irrigant as a result of activating the irrigation solution with different systems. The null hypothesis was rejected.

The protocol for this study was designed to maximize the possibility of extrusion of irrigating solution through an unrestricted, yet normal apex. To standardize the apical constriction, apical patency was checked with a size 10-K file between each instrument. All control and activated groups were irrigated from 2 mm short of the working length with 4 mL NaOCl using 27- gauge needle for obtaining a standardization of irrigation. In laser groups the fiber tip was inserted 1 mm short of the working length as the manufacturers’ instructions. In PUI group the file was also inserted 1 mm short of the working length to obtain standardization between activations. In the present study, each tooth was secured through the lid of an Eppendorf tube filled with distilled water to collect the apically extruded irrigating solution. The amount of extruded NaOCl in the Eppendorf tubes was determined by the chemical reaction of OCl^−^ with I^−^ (iodide) in an acidic solution. Following the chemical reaction, the amount of NaOCl was determined by spectrophotometric assessment of the change in color due to the formation of iodine (I_2_) in aqueous solution which is consistent for detecting small increments of extruded material. Various methods have been used to evaluate apical extrusion of irriganting solutions *in vitro,* however this model provides sensitivity along with quantification of extrusion of irrigating solution [24, 25].

The results of the current study revealed that apical extrusion increased with the activation of sodium hypochlorite irrigation. Non-activated syringe irrigation showed less apical extrusion of irrigating solution than activated methods. Meire et al. [26] found that the absorption coefficient values of NaOCl at 980 nm diode laser and 1064 nm Nd:YAG laser are 0.250 and 0.108 cm^−1^ respectively. These values are well below the absorbtion coefficient values required for occurrence of cavitation at the laser tip (10 cm^−1^). However, the absorption coefficient of the irrigation solution is not the only factor that effects the occurance of cavitation at the laser fiber tip. The wavelength, power density and energy density also play a role. It has been suggested that 940-nm and 980-nm diode lasers also are able to induce cavitations and hence activate irrigants [22]. Lauterborn and Ohl [27] produced cavitation bubbles in water using a laser with very low absorption in water (Nd:YAG laser; 1.064 nm alpha of 0.1 cm^−1^ 98 % transmission through 1 mm). In this study although apical extrusion occurred with both lasers in different modes, the Nd:YAG laser activation showed a greater amount of extrusion of irrigating solution. As the lasers were used in accordance with the manufacturers’ instructions, it may be related to high power setting of Nd:YAG laser. In this study, diode laser was used in a continuous wave, whereas the Nd:YAG laser was used in pulsed mode. Due to the pulsations of the laser, the fluid becomes accelerated at every pulse and the acceleration gives rise to the inertia forces [28]. Pulsed lasers can create pressure waves of sufficient force to propel microdroplets of the aqueous irrigating solution beyond the apical constriction [18]. Thus, caution should be exercised when using such lasers in combination with irrigating solutions such as sodium hypochlorite.

The PUI group also showed apical extrusion of irrigating solution. One of the previous studies showed that systems using ultrasound cleaned the endodontic space more effectively; however, that cleansing action was more difficult to control [29] . There might be a risk of pushing the irrigating solution beyond the apex. The PUI system showed absolute safety when used at 5 and 3 mm, whereas there was some extrusion of NaOCl in almost all tests at 1 mm [29]. The result of this study, contradicts an earlier report [30] which concluded that no significant difference was detected between PUI techniques and passive extrusion (control) of irrigating solution. The application time (10 s) of PUI differs from this study (60s). In a spectrophotometric study, Rodriguez-Figueroa et al. [25] used PUI 2 mm short from the working length, and concluded that PUI as a safe device. However, in this study we used PUI 1 mm short from the working length.

## Conclusion

Within the limitations of this *in vitro* study, the researchers concluded that non-activated syringe irrigation caused less apical extrusion of irrigating solution than PUI and LAI using Nd:YAG or diode lasers..

## Abbreviations

PUI: Passive ultrasonic irrigation
LAI: Laser-activated irrigation
NaOCl: Sodium hypochlorite
Nd:YAG laser: Neodymium-doped yttrium aluminium garnet laser
I_2_: Iodine
